# Trends of loss of peripheral muscle thickness on ultrasonography and its relationship with outcomes among patients with sepsis

**DOI:** 10.1186/s40560-018-0350-4

**Published:** 2018-12-12

**Authors:** Vijay Hadda, Rohit Kumar, Gopi Chand Khilnani, Mani Kalaivani, Karan Madan, Pawan Tiwari, Saurabh Mittal, Anant Mohan, Ashu Seith Bhalla, Randeep Guleria

**Affiliations:** 1 Department of Pulmonary Medicine and Sleep Disorders, All India Institute of Medical Sciences, New Delhi, India; 2 Department of Biostatistics, All India Institute of Medical Sciences, New Delhi, India; 3 Department of Radio-diagnosis, All India Institute of Medical Sciences, New Delhi, India

**Keywords:** Ultrasonography, Muscle thickness, Critical illness, ICU, Sepsis, Outcome

## Abstract

**Background and aims:**

Data regarding trends of muscle loss on ultrasonography (USG) and its relationship with various outcomes among critically ill patients is limited. This study aimed to describe the trends of loss of muscle thickness of the arm and thigh (assessed using USG) and to determine the relationship between loss of muscle thickness and in-hospital and post-discharge outcomes.

**Methods:**

Muscle thickness of 70 patients with sepsis was measured at the level of the mid-arm and mid-thigh using bedside USG on days 1, 3, 5, 7, 10 and 14 and then weekly till discharge or death. Patients were followed up for 90 days after discharge.

**Results:**

The muscle thickness (mean ± SD) at the level of the mid-arm and mid-thigh on day 1 was 23.13 ± 4.83 mm and 31.21 ± 8.56 mm, respectively. The percentage muscle thickness [median (min, max)] decline at the mid-arm and mid-thigh was 7.61 (− 1.51, 32.05)% and 10.62 (− 1.48, 32.06)%, respectively on day 7 as compared to baseline (*p* < 0.001). The decline in muscle thickness at the mid-arm and mid-thigh were higher among non-survivors compared to survivors at all time points. Also, the decline in muscle thickness was significantly higher among patients with worse outcome at day 90. Patients with ICU-acquired weakness also had significantly higher decline in muscle thickness (*p* < 0.05). Early decline (from day 1 to day 3) in muscle thickness was associated with in-hospital mortality. The probability of death by day 14 was higher for patients who had early decline (from day 1 to day 3) in muscle thickness of ≥ 6.59% and ≥ 5.20% at the mid-arm [HR 7.3 (95% CI 1.5, 34.2)] and the mid-thigh [HR 8.1 (95% CI 1.7, 37.9)], respectively. Decline in thickness from day 1 to day 3 was a good predictor of in-hospital mortality with area under the curve (AUC) of 0.81 and 0.86 for arm and thigh muscles, respectively.

**Conclusions:**

Critically ill patients with sepsis exhibit a gradual decline in muscle thickness of both the arm and thigh. Decline in muscle thickness was associated with in-hospital mortality. USG has a potential to identify patients at risk of worse in-hospital and post-discharge outcomes.

**Electronic supplementary material:**

The online version of this article (10.1186/s40560-018-0350-4) contains supplementary material, which is available to authorized users.

## Background

Weakness of skeletal muscles is among the major risk factors associated with increased duration of stay in intensive care units (ICU) and hospital, in-hospital mortality and physical disability [1, 2]. This weakness is a result of muscle wasting due to immobility, sepsis, organ dysfunction, drugs and systemic inflammation [3]. Critically ill patients with sepsis are at risk of developing muscle wasting [4]. However, accurate quantification of muscle wasting remains a challenge among these patients [5]. Assessment of muscle wasting using clinical examination and anthropometry has limitations in this setting [1]. Imaging of these muscles with computer tomographic (CT) scan, magnetic resonance imaging (MRI), or dual energy X-ray absorptiometry (DEXA) scan is though accurate but not practical in patients admitted to ICU [1]. Bioelectrical impedance analysis can also be used to estimate muscle mass; however, it can be affected by the variable hydration status and requires special apparatus. Therefore, a sensitive, reliable and safe tool for measurement of loss of muscle objectively is required.

Ultrasonography (USG) has been used as a tool for assessment of muscle thickness in ICU setting [4, 6, 7]. Muscle thickness has been shown as a good surrogate of muscle mass and can be measured using USG with excellent intra- and inter-observer reliability [6, 8, 9]. Non-invasive nature and bedside availability makes USG an ideal tool for serial assessment of thickness of peripheral muscles. Serial assessment can provide useful information regarding the muscle loss in relation to time among critically ill patients in ICU. However, majority of published studies on assessment of muscle thickness in ICU are cross-sectional in design [7]. There are only few studies which have used USG for measurement of muscle thickness at standardized time points for comparison [10–12]. Muscle dysfunction assessed by various tools has been associated with both in-hospital as well as post-discharge mortality and morbidity [1, 2]. USG can be used for serial assessment of muscle dysfunction; however, there is a lack of data regarding relationship between change in muscle thickness measured by USG and in-hospital and post hospital discharge outcomes.

We planned this prospective study to evaluate the trends of change in muscle thickness of the arm and thigh among critically ill patients with sepsis. We also sought to describe the relationship between change in muscle thickness and in-hospital and day-90 outcome among these patients.

## Materials and methods

### Study design, patients and setting

This prospective study was conducted between March 2015 and December 2016 at a tertiary care teaching hospital. All adult (age ≥ 18 years) patients admitted under Pulmonary Medicine services with a diagnosis of sepsis with medical illness (non-surgical) were eligible for inclusion in the study. The diagnosis of sepsis was based on the criteria proposed by SCCM/ESICM/ACCP/ATS/SIS International Sepsis Definitions Conference [13]. Criteria included the presence of infection (proven or suspected) *plus* presence of systemic inflammatory response syndrome (SIRS). SIRS may be recognized by the presence of any one of the following (but limited to): temperature more than 38 °C or less than 36 °C, heart rate > 90/min, respiratory rate > 30/min, altered mental status, positive fluid balance, leukocytosis or leukopenia, more than 10% of immature neutrophils and increased C-reactive protein or procalcitonin. Patients with any known neuro-muscular diseases such as myopathy, neuropathy, or stroke; with dressing over or imputed right limb; with recent (within 3 months) hospitalization and need of ventilator support or home ventilation; who transferred from other hospital after stay of more than 24 h; and who refused to give consent for the study were excluded. Also, patients with duration of hospital stay less than 72 h (due to death or discharge) were excluded from the study.

### Equipment and operator

For this study, we used Siemens ACUSON X300™ (Siemens Health Care, Germany) machine for measurement of the muscle thickness. Muscle thickness measurements were done using B-mode of USG using 5.0–13.0 MHz (megahertz) linear array probe (VF 13-5). All measurements were taken by a single operator (RK) who was trained in using bedside USG in ICU.

### Site, posture and measurement technique

Measurements of muscle thickness were done on the right side, at the level of the mid-arm and mid-thigh. All measurements were done in supine position, unless contra-indicated. For the arm, the flexor compartment (biceps brachii and coracobrachialis) and, for thigh, the extensor compartment (quadriceps muscles) of muscles were selected. The measurements of the muscle thickness of the flexor compartment of the arm and quadriceps muscles were performed following similar protocol used by Campbell and colleagues in 1997 and recently validated by us [6, 8, 9]. Briefly, a circumferential mark was applied at the midway between the greater tuberosity and the tip of the olecranon process of the humerus. Similarly, a circumferential mark was applied at the midway between the tip of the greater trochanter and the lateral joint line of the knee. The linear array USG probe was placed on the anterior aspect of this circumferential line, perpendicular to the skin, and the probe was moved along the line drawn till a suitable image was obtained. Keeping the focus on the suitable image, a point corresponding to the centre of the probe was marked with a vertical line. Same procedure was done for the arm and thigh. This vertical point on the circumferential line was used as the reference point for all subsequent measurements.

Arm muscle thickness was measured while the upper limb relaxed, lying parallel to the body, the elbow extended and the palm facing the ceiling. Thigh muscle thickness was measured while the leg resting straight, the knee extended and the great toe facing the ceiling. The measurements of the muscle thickness was done using built-in callipers on the real-time frozen image (Additional file 1).

### Muscle thickness measurement schedule

The measurements of the muscle thickness at both the sites were recorded on days1, 3, 5, 7, 10 and 14 and subsequently on a weekly basis, till discharge or death of the patient.

### Outcome measurement

In-hospital outcome parameters including length of stay (LOS) in the hospital and ICU, time spent on ventilator and death or survival were recorded. Whenever the patients were awake and cooperative during the time of the USG examination, a clinical assessment of the muscle strength was also done using Medical Research Council (MRC) sum score. Patients with an MRC sum score of < 48 were classified as having ICU-acquired weakness (ICU-AW) [14]. Post discharge, survivors were contacted telephonically on day 90. They were asked regarding worsening of condition requiring unscheduled visit to hospital or emergency or hospitalization. For the analysis of 90-day outcome, death or worsening of condition leading to unscheduled visit to hospital or emergency or hospitalization was labelled as poor outcome and survivors without any of these were classified as stable outcome.

### Statistical analysis

The sample size of 70 was based only on feasibility. Data analysis was performed with Stata14.0 (StataCorp LP, College Station, TX 77845, USA). Measurements taken till day 7 (days 1, 3, 5 and 7) and last measurement (irrespective of day) were included in the final analysis as there were only 16, 11, 3 and 2 patients on days 10, 14, 21 and 28, respectively. Data were expressed as number (%) or mean ± standard deviation (SD) or median with inter-quartile range (IQR) as deemed appropriate. We used marginal model of Generalized Estimating Equation (GEE) for assessing the magnitude of change in the muscle thickness at each time point (days 1, 3, 5 and 7) and also to show whether loss of muscle thickness was more during early or later part of hospital stay. Percentage loss of thickness was compared between two groups of various outcomes such as in-hospital mortality, ICU-AW and 90-day outcome at each time points using Wilcoxon rank sum test since the data was not normally distributed.

Receiver operating characteristics (ROC) curve analysis was used to compare the predictive ability of loss (%) of muscle thickness of the arm and thigh (at day 3 with respect to day 1) and Acute Physiology and Chronic Health Evaluation (APACHE)-II and Sequential Organ Failure Assessment (SOFA) (at day 3) scores against in-hospital mortality. ROC analysis showed that a cutoff of decline in muscle thickness of more than or equal to 6.59% for the arm and 5.20% for the thigh can predict survival. Kaplan-Meier curve was used to depict probability of survival over time between the categories (derived by ROC analysis) of decline in percent muscle thickness of the arm and thigh followed by log-rank test to compare these survival probabilities. Cox proportional hazards model was used to calculate the hazards of mortality (95% CI) associated with decline in percent muscle thickness of the arm and thigh of ≥ 6.59% and ≥ 5.20%, respectively. Logistic regression analysis was used to find the odds associated with decline in percent muscle thickness of the arm and thigh of ≥ 6.59% and ≥ 5.20%, respectively, on prolonged (> 2 weeks) mechanical ventilation and ICU stay. The *p* value of < 0.05 was considered as statistically significant.

### Ethical aspect

The study was conducted following guidelines for biomedical research involving human subjects [15, 16]. Study protocol was approved by Institutional Ethics Committee.

The manuscript is written according to STROBE statement for observation studies.

## Results

**Fig. 1:**
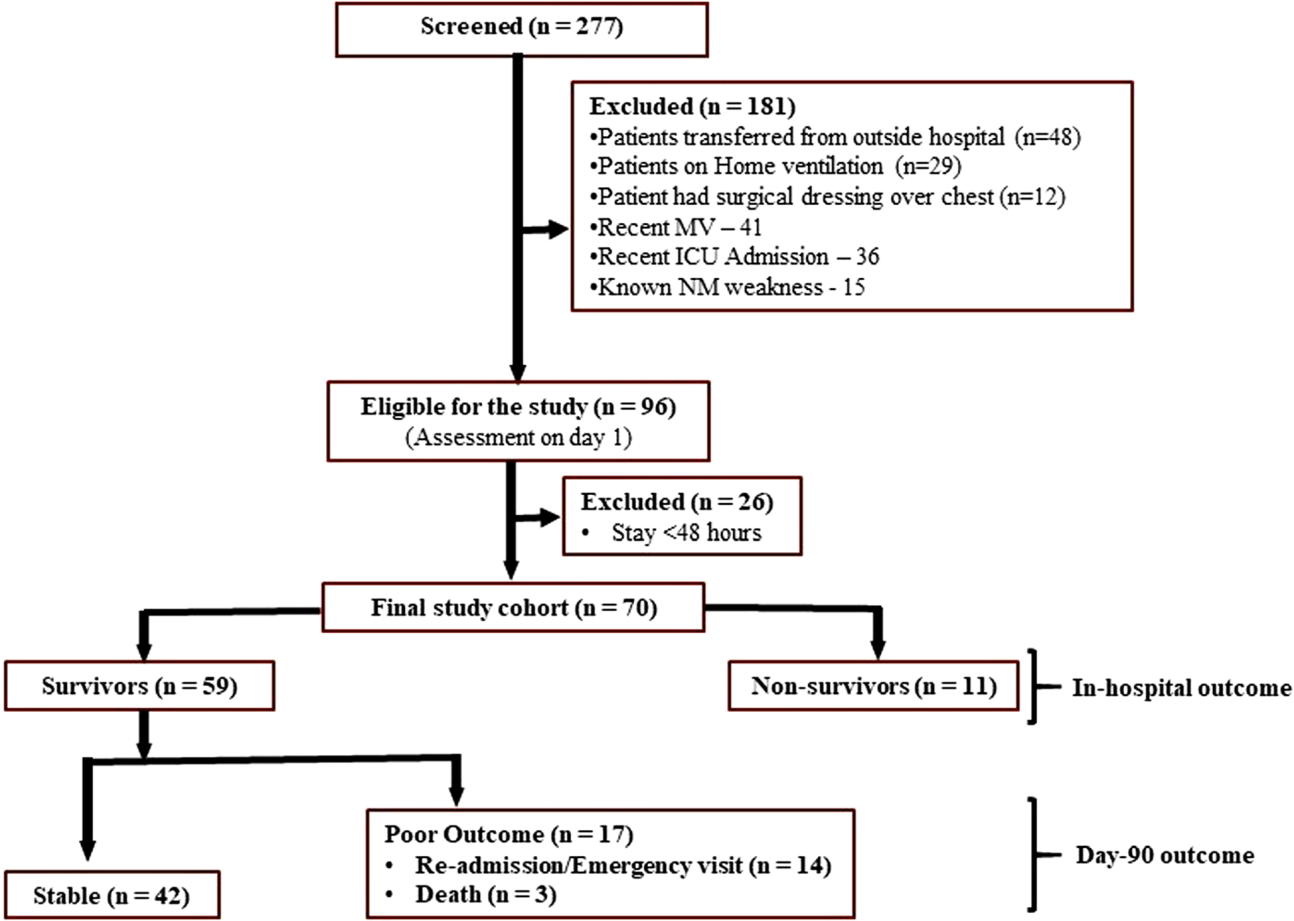
The flow of participants in the study

There were 96 eligible patients who were evaluated on day 1. Among these, 26 (19 died and 7 discharged) patients were excluded before evaluation on day 3. The final study cohort included 70 (45 male) patients admitted with sepsis. The flow of participants in the study is shown in Fig. 1. Study patients had severe illness with APACHE (mean ± SD) and SOFA (mean ± SD) scores of 17.17 ± 4.42 and 5.71 ± 2.05, respectively. The baseline features of the study cohort are summarized in Table 1.

**Table 1:** Baseline characteristics of the study cohort

Variables	Observation (*N* = 70)
Age (mean ± SD, years)	55.91 ± 14.08
Gender	
Male (%)	45 (64.3%)
APACHE-II score (mean ± SD)	17.17 ± 4.42
SOFA score (mean ± SD, day 1)	5.71 ± 2.05
Height (mean ± SD, cm)	158.45 ± 5.89
Haemoglobin (mean ± SD, gm/dl)	12.29 ± 2.07
Haematocrit (mean ± SD, gm/dl)	39.55 ± 7.32
Leukocyte counts (mean ± SD, per dl)	13,799 ± 4998
Blood Urea [median (interquartile range), mg/dl]	54 (13–191)
Serum creatinine [median (interquartile range), mg/dl]	0.9 (0.3–4.7)
Serum bilirubin [median (interquartile range), mg/dl]	0.6 (0.2–3.7)
Serum albumin (mean ± SD, gm/dl)	3.17 ± 0.49
Serum calcium (mean ± SD, gm/dl)	8.02 ± 0.76
Serum phosphate (mean ± SD, gm/dl)	3.27 ± 1.12
Anterior arm muscle thickness (mean ± SD, mm)	23.13 ± 4.83
Quadriceps muscle thickness (mean ± SD, mm)	31.21 ± 8.56
Source of infection	
Lower respiratory tract	67(95.7%)
Urinary tract	3(4.3%)
Co-morbidities	
COPD	40(57%)
Asthma	7(10%)
Bronchiectasis	7(10%)
Interstitial lung diseases	2(3%)

### Trends of muscle thickness on USG

**Fig. 2:**
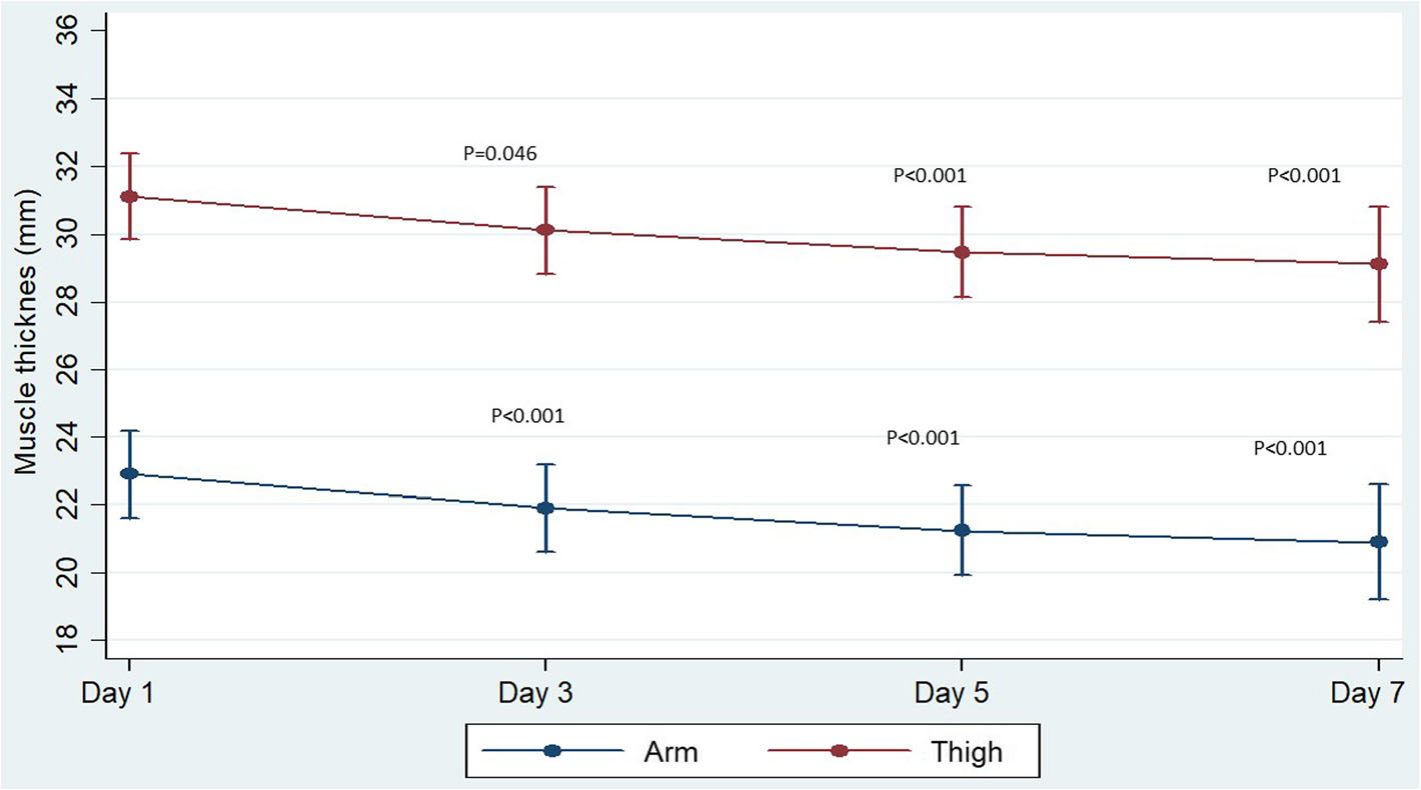
Trend in muscle thickness in mm (both the arm and thigh) with 95% CI over days 1, 3, 5 and 7 during hospital stay

The muscle thickness (mean ± SD) measured at the level of the mid-arm and mid-thigh on day 1 was 23.13 ± 4.83 mm and 31.21 ± 8.56 mm, respectively. The muscle thickness (%), both at mid-arm and mid-thigh levels, showed a significant decline at each time points of assessment (days 3, 5 and 7 and last assessment) with respect to baseline (Fig. 2).

### Loss of muscle thickness and outcomes

- In-hospital mortality

Among the study cohort, there were 11 deaths during hospital stay. At day 1, the mean thickness of arm and thigh muscles was comparable between survivors and non-survivors (Tables 2 and 3). As shown in Tables 2 and 3, the percentage decline in the muscle thickness of the arm and thigh was higher among non-survivors as compared to survivors at all time-points of assessment.

**Table 2:** Decline in arm muscle thickness and outcome

Muscle thickness	Day 1	Day 3	Day 5	Day 7
a. In-hospital mortality				
Survivors (*n*)	59	59	53	28
Muscle thickness (mean ± SD)	23.0 ± 5.4	22.5 ± 4.9	21.9 ± 4.9	21.0 ± 5.3
% Decline [median (IQR)]	NA	3.5 (1.3, 5.8)	5.5 (2.8, 8.8)	4.7 (2.7, 12.1)
Non-Survivors (*n*)	11	11	10	7
Muscle thickness (mean ± SD)	21.6 ± 4.1	19.8 ± 3.7	19.0 ± 2.9	18.0 ± 2.5
% Decline [median (IQR)]	NA	8.8 (6.7, 10.1)	15.5 (13.1, 16.4)	19.6 (10.7, 21.7)
*p* value (% decline)	NA	0.001	< 0.001	< 0.013
b. ICU-acquired weakness				
Absent (*n*)	55	55	48	23
Muscle thickness (mean ± SD)	22.4 ± 5.1	22.0 ± 4.5	21.5 ± 4.5	20.5 ± 4.2
% Decline [median (IQR)]	NA	3.6 (1.3, 5.8)	5.6 (2.8, 9.5)	5.6 (2.5, 11.2)
Present (*n*)	15	15	15	12
Muscle thickness (mean ± SD)	24.0 ± 5.9	22.4 ± 5.8	21.4 ± 5.9	20.2 ± 6.4
% Decline [median (IQR)]	NA	6.5 (2.7, 9.6)	11.8 (3.2, 17.1)	16.8 (4.3, 25.7)
*p* value (% decline)	NA	0.01	0.05	0.03
c. 90-day outcome				
Stable outcome (*n*)	42	42	36	17
Muscle thickness (mean ± SD)	23.3 ± 5.6	23.3 ± 4.8	23.1 ± 4.8	23.0 ± 5.2
% Decline [median (IQR)]	NA	2.3 (1.0, 3.8)	3.6 (2.6, 5.8)	4.0 (2.1, 4.4)
Poor outcome (*n*)	17	17	17	11
Muscle thickness (mean ± SD)	22.2 ± 5.1	20.5 ± 4.6	19.3 ± 4.4	18.0 ± 3.9
% Decline [median (IQR)]	NA	8.6 (5.9, 11.1)	13.4 (8.8, 17.1)	14.4 (11.2, 17.1)
*p* value (% decline)	NA	< 0.0001	< 0.0001	0.0004

**Table 3:** Decline in thigh muscle thickness and outcome

Muscle thickness	Day1	Day3	Day5	Day7
a. In-hospital mortality				
Survivors (*n*)	59	59	53	28
Muscle thickness (mean ± SD)	31.3 ± 8.5	30.2 ± 8.3	29.6 ± 8.0	30.4 ± 7.4
% Decline [median (IQR)]	NA	2.6(1.4,5.1)	3.7(2.8, 8.3)	4.8(3.6, 9.8)
Non-Survivors (*n*)	11	11	10	7
Muscle thickness (mean ± SD)	30.9 ± 9.4	28.5 ± 9.0	27.6 ± 8.7	26.4 ± 8.7
% Decline [median (IQR)]	NA	7.5(5.2, 11.4)	13.0(10.0, 15.7)	15.5(10.4, 17.8)
*p* value (% decline)	NA	< 0.001	< 0.001	< 0.003
b. ICU-acquired weakness				
Absent (*n*)	55	55	48	23
Muscle thickness (mean ± SD)	31.5 ± 8.1	30.3 ± 7.8	29.9 ± 7.2	29.9 ± 6.8
% Decline [median (IQR)]	NA	2.9 (1.6, 5.2)	3.7 (2.9, 9.1)	5.1 (3.3, 10.4)
Present (*n*)	15	15	15	12
Muscle thickness (mean ± SD)	30.2 ± 10.3	28.5 ± 10.4	27.3 ± 10.4	28.9 ± 9.5
% Decline [median (IQR)]	NA	5.7 (2.9, 8.4)	9.9 (5.5, 13.0)	11.0 (6.5, 16.8)
*p* value (% decline)	NA	0.04	0.02	0.02
c. 90-days outcome				
Stable outcome (*n*)	42	42	36	17
Muscle thickness (mean ± SD)	31.7 ± 7.9	31.0 ± 7.7	30.9 ± 7.0	31.6 ± 6.6
% Decline [median (IQR)]	NA	2.1 (− 5.0, 7.9)	3.1 (− 2.0, 9.6)	4.2 (− 2.6, 9.1)
Poor outcome (*n*)	17	17	17	11
Muscle thickness (mean ± SD)	30.1 ± 9.9	28.2 ± 9.6	26.8 ± 9.3	28.5 ± 8.6
% Decline [median (IQR)]	NA	5.7 (0.8, 14.4)	9.9 (3.5, 26.9)	10.9 (2.7, 33.7)
*p* value (% decline)	NA	< 0.0001	< 0.0001	0.0003

**Fig. 3:**
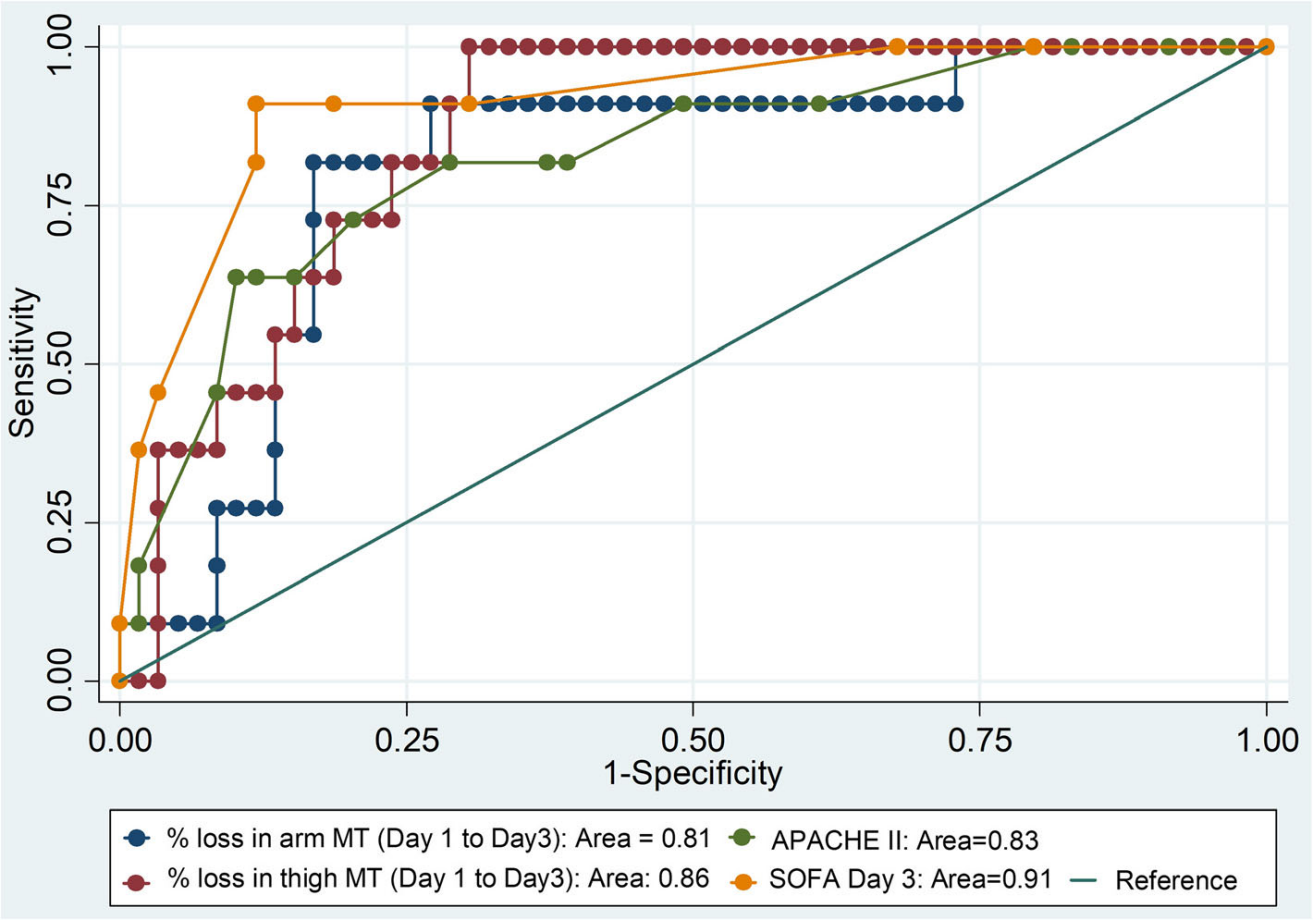
ROC analysis showing comparison of in-hospital survival predictive ability of percent loss in arm and thigh muscle thickness from days 1 to 3, APACHE score on day 1 and SOFA score on day 3

**Fig. 4:**
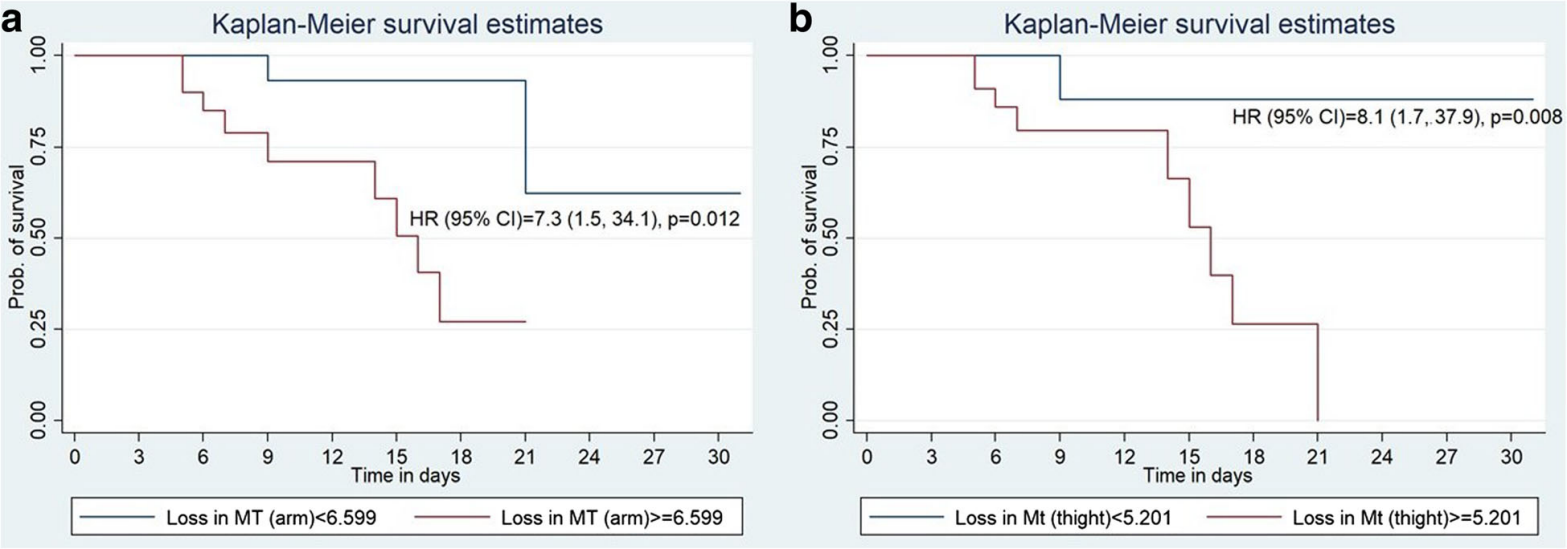
Kaplan-Meier curve showing probability of survival over 28 days of hospital stay between the two groups of loss in muscle thickness of the arm (**a**) and the thigh (**b**)

ROC analysis showed that a cutoff of decline in muscle thickness of more than or equal to 6.59% for the arm and 5.20% for the thigh can predict survival (Fig. 3). As shown in Fig. 4a and b, the probability of survival (95% CI) at day 14 was higher with < 6.59% [93.3% (61.3%, 99.0%)] than ≥ 6.59% [60.9% (30.4%, 81.3%)] of loss of arm muscle thickness; the hazard of dying with loss of arm muscle thickness of ≥ 6.59% was 7.3 times (95% CI 1.5–34.2) higher than their counterparts with *p* value 0.012. The probability of survival (95% CI) at day 14 with the loss of thigh muscle thickness of < 5.20% and ≥ 5.20% was 88.2% (60.6%, 96.9%) and 66.3% (31.4%, 86.4%), respectively; *p* value < 0.002. ROC analysis also revealed that SOFA score was the best predictive tool (shown in Table 4 and Fig. 3) for in-hospital mortality followed by the loss of thigh muscle thickness between day 1 and day 3.

b. Requirement of mechanical ventilation and stay in ICU

**Table 4:** ROC analysis for in-hospital mortality predictive ability of muscle thickness (MT), APACHE II and SOFA

Variables	Cutoff	Sensitivity	Specificity	LR	Area (95% CI)
Loss of arm MT between days 1 and 3 of measurement (%)	6.59	81.8	81.4	4.4	0.81 (0.68, 0.94)
Loss of thigh MT from days 1 to 3(%)	5.20	81.8	76.3	3.4	0.86 (0.77, 0.95)
APACHE II at admission	19	81.8	71.2	2.8	0.83 (0.69, 0.96)
SOFA on the day 3	5	90.9	88.1	7.7	0.91 (0.82, 1.0)

Total duration of ICU stay and requirement of mechanical ventilation ranged from 3 to 31 days and 0 to 31 days, respectively. The mean (± SD) of ICU stay and time spent on mechanical ventilation were 7.24 (±5.1) days and 3.9 (±5.3) days, respectively. Odds ratio (95% CI) for requirement of prolonged (≥ 2 weeks) mechanical ventilation associated with the decline of ≥ 6.59% and ≥ 5.20% of arm and thigh muscle thicknesses was 4.2(0.65, 27.6), *p* = 0.131, and 9.7(1.01, 92.4), *p* = 0.049, respectively. Odds ratio (95% CI) for prolonged (≥ 2 weeks) ICU stay associated with muscle thickness of ≥ 6.59% and ≥ 5.20% of arm and thigh muscle thicknesses was 3.8 (0.90, 16.1), *p* = 0.067, and 3.0 (0.72, 12.4), *p* = 0.132, respectively.

c. ICU-acquired muscle weakness

ICU-AW was diagnosed in 15 patients. The comparisons of loss of muscle thickness was done between patients with and without ICU-AW (MRC score < 48 vs ≥ 48), at different time points (Tables 2 and 3). Median (IQR) percentage decline in muscle thickness of the arm and thigh at day 7 was higher for those who had ICU-AW than those without it.

d. Outcome during 90 days follow-up

During 90 days follow-up, 17 out of 59 survivors had poor outcome (death—3, emergency visit or re-admission—14) whereas 42 remained stable. The decline in muscle thickness, both the arm and thigh, was higher in patients with poor outcome than in those with stable outcome during 90 days follow-up, which is shown in Tables 2 and 3.

## Discussion

This study showed that USG can be used for demonstration of trends of loss of arm and thigh muscles thickness among patients with sepsis during ICU and hospital stay. Our results demonstrated that patients with sepsis lose approximately 9–10% of muscle thickness during hospital stay. The loss of muscle thickness was significantly more among patients who died during hospital stay or had adverse outcomes during 90 days following discharge from hospital.

Critically ill patients with sepsis are at increased risk of loss of muscle mass [4, 17]. Demonstration of trends of the muscle loss is desirable for appropriate risk stratification as well as for any preventive or therapeutic intervention. There are few other studies which have reported the loss of muscle thickness on USG among critically ill patients [10–12]. Our study results further highlighted that loss of muscle is common among critically ill patients and the loss is the greatest during early days of hospitalization (3 days). Therefore, any preventive intervention for muscle wasting should be started at the earliest, probably soon after admission.

The muscle dysfunction has been shown to be an independent risk factor for in-hospital mortality [1, 18, 19]. However, none of the previously published studies on muscle thickness on USG reported the data related to muscle thickness and outcomes [10–12]. Probably, this is the first study that has reported the data regarding the relationship between loss of muscle thickness and in-hospital and post-discharge outcomes. Importantly, the decline in muscle thickness on USG was able to differentiate survivors from non-survivors as early as day 3 of hospitalization. Among critically ill patients in addition to muscle loss, multiple factors including age and gender of the patient, severity of illness, standard of care and associated co-morbidities which may affect the survival [20, 21]. However, these results provided us the objective values of muscle thickness which may be useful in the identification of patients who may be at risk of worse in-hospital and 90-day outcomes. Also, this study provided the reference values of muscle thickness for calculation of sample size for any future preventive or therapeutic intervention for the management of muscle dysfunction among patients with sepsis.

Application of MRC score for the diagnosis of ICU-AW is limited by feasibility as well as reliability [22–24]. Our study has demonstrated the relationship between declines in muscle thickness with ICU-AW. These results suggest that USG may be a better tool for early and timely identification of individuals at risk of development of ICU-AW.

This is one of the largest prospective studies demonstrating the feasibility of USG for the assessment of the muscle thickness over time. Also, to the best of our knowledge, this is the first study which has described the relationship between decline in arm and thigh muscle thickness on USG and various in-hospital and post discharge outcomes among critically ill patients with sepsis. We recognize that there are many limitations of this study. First, the sample size was determined based on feasibility. Therefore, the study may not be powered enough to detect an independent relationship between the decline in the muscle thickness and various in-hospital and post-discharge outcomes assessed. Also, the number of study participants decreased significantly after day 7 of recruitment leaving few patients for any meaningful analysis, though, this loss of study participants was not in our control as the reasons for this was either death or discharge. It was a single-centre study, and image acquisition and measurements were done by experienced operator; therefore, one may question its wider application. Measurement of muscle thickness on USG is considered as operator-dependent, and one may question the validity of the measurements. In our experience, inter- and intra-observer reliability for measurements of muscle thickness of the arm and thigh on USG was excellent [8, 9, 25]. It is suggested that before using USG for this purpose, reliability of the measurements must be checked. We could not do echogenicity analysis because of lack of the software required for this. We did not compare the measurement of thickness on USG with that of thickness measured by other standard tool such MRI, CT scan, or fluoroscopy. Such comparisons are desirable for the assessment of accuracy; however, these are not practical in ICU setting. However, muscle thickness measured using USG correlates well with measurements done with these tools [26]. The muscle biopsy is gold standard for the demonstration of muscle loss. Therefore, demonstration of correlation of muscle thickness on USG and biopsy would have been the best as shown by Puthucheary and colleagues in a landmark study [27]. However, we did not perform muscle biopsy due to its invasive nature. Regardless of inclusion criteria of sepsis, the included patients were more serious with severe sepsis or septic shock. It was probably the result of limited availability of beds due to which only the sickest patients could be admitted. There are certain drugs such as muscle relaxants, corticosteroids, sedatives and aminoglycosides which may affect the muscle functions. We did not record these parameters as our primary objective was to describe the trends, not the predictors of muscle loss. Hence, effects of these drugs on muscle thickness among these patients could not be commented. Also, we should recognize that the presence of many confounder such as age and gender of the patient, severity of illness, standard of care and associated co-morbidities can affect the both muscle thickness as well as outcomes; however, we could not perform analysis to adjust these due to limited number of events.

## Conclusions

We conclude that USG is a useful tool for serial assessment of muscle thickness in ICU settings. It has a potential to identify the patients at risk of development of ICU-AW, in-hospital mortality and 90-day adverse outcome within a few days of hospitalization.

## Additional file

Additional file 1: **Figure S1.** Showing real-time frozen image for measurement of the arm muscle thickness of the arm (both biceps and coracobrachialis muscle may be seen). The thickness is measured between superficial fat-muscle interface and periosteum. Figure S2. Measurement of thigh muscle thickness. Figure S3. The marking over the thigh and arm. Figure S4. Kaplan Meier survival curve for change in muscle thickness between days 1 and 3. (DOCX 592 kb)

## Abbreviations

AUC: Area under the curve
USG: Ultrasonography
SD: Standard deviation
ICU: Intensive care unit
HR: Hazard ratio
CI: Confidence intervals
CT: Computer tomography
MRI: Magnetic resonance imaging
DEXA: Dual energy X-ray absorptiometry
LOS: Length of stay
ICU-AW: ICU-acquired weakness
MRC: Medical Research Council
ROC: Receiver operating characteristics
APACHE: Acute Physiology and Chronic Health Evaluation
SOFA: Sequential Organ Failure Assessment
