# Supplementary material for: Trends of loss of peripheral muscle thickness on ultrasonography and its relationship with outcomes among patients with sepsis
Source: J Intensive Care. 2018 Dec 12;6:81. doi: 10.1186/s40560-018-0350-4 (PMC6292013; doi:10.1186/s40560-018-0350-4)

**Supplementary Material**

**Title:** Trends of loss of peripheral muscle thickness on ultrasonography and its relationship with outcomes among patients with sepsis

**Figure S1.** Showing real time frozen image for measurement of the arm muscle thickness of arm (both biceps and coracobrachialis muscle may be seen). The thickness is measured between superficial fat-muscle interface and periosteum.

**
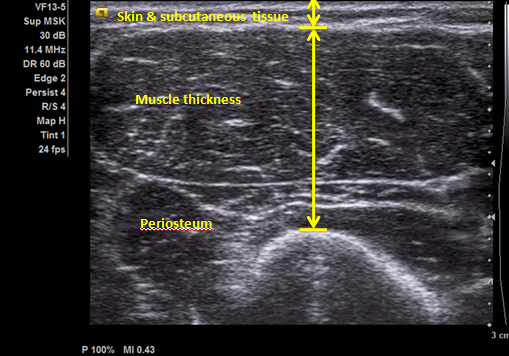
**

**Figure S2. Measurement of thigh muscle thickness**

**
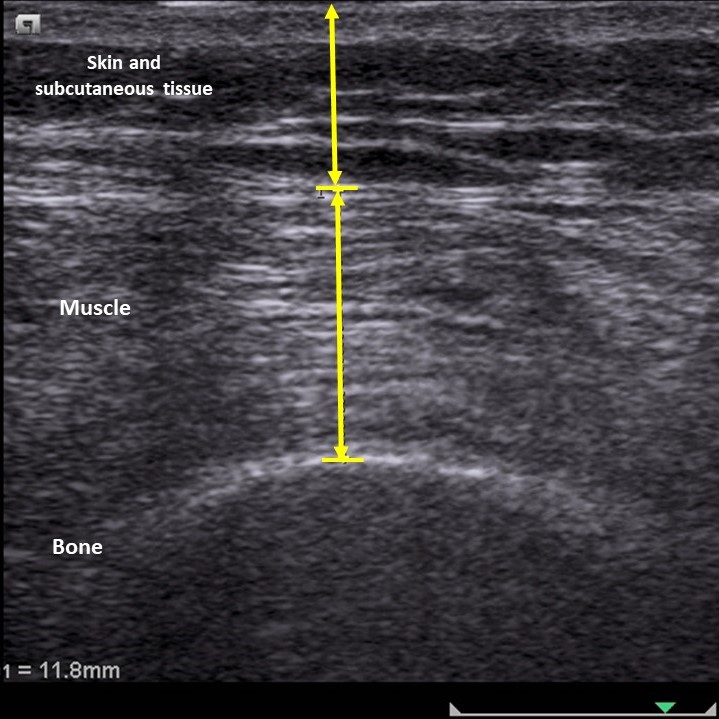
**

**
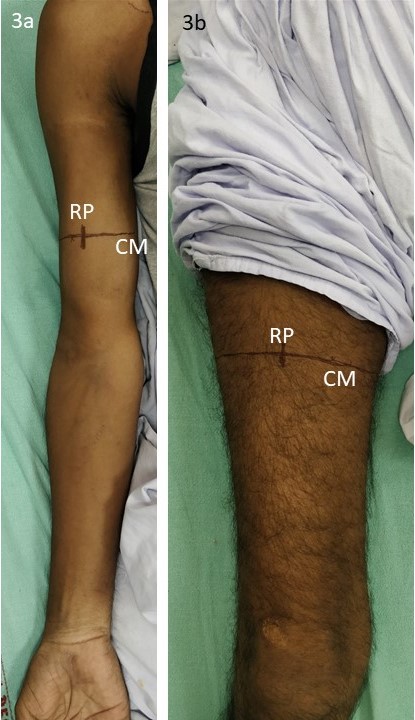
**

**Figure S3.** The marking over the thigh and arm. **A** circumferential mark (CM) was applied at the midway between the greater tuberosity and the tip of the olecranon process of humerus (Fig 3a). Similarly, a circumferential mark (CM) was applied at the midway between the tip of the greater trochanter and the lateral joint line of the knee (fig 3b). The linear array USG probe was placed on the anterior aspect of this circumferential line, perpendicular to the skin and the probe was moved along the line drawn till a suitable image was obtained. Keeping the focus on the suitable image, a point corresponding to the center of the probe was marked with a vertical line and this point was taken as reference point (RP) for all the subsequent measurements.

Figure S4. Kaplan Meier survival curve for change in muscle thickness between day 1 and 3


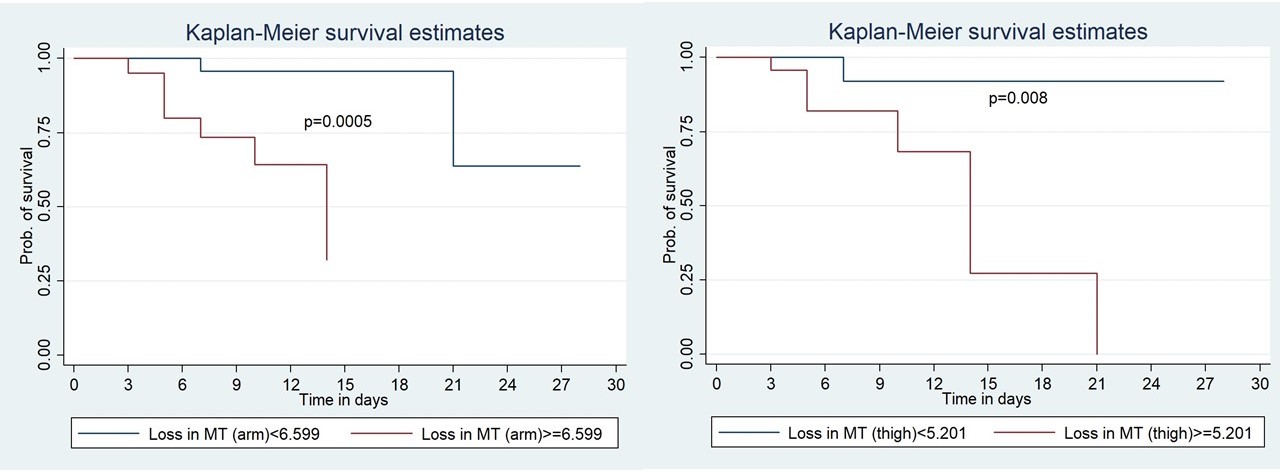

Supplement: Supplementary file 1 — Figure S1. Showing real-time frozen image for measurement of the arm muscle thickness of the arm (both biceps and coracobrachialis muscle may be seen). The thickness is measured between superficial fat-muscle interface and periosteum. Figure S2. Measurement of thigh muscle thickness. Figure S3. The marking over the thigh and arm. Figure S4. Kaplan Meier survival curve for change in muscle thickness between days 1 and 3. (DOCX 592 kb) [file 40560_2018_350_MOESM1_ESM.docx]
